# The Dark Side of Humor: DSM-5 Pathological Personality Traits and Humor Styles

**DOI:** 10.5964/ejop.v12i3.1109

**Published:** 2016-08-19

**Authors:** Virgil Zeigler-Hill, Gillian A. McCabe, Jennifer K. Vrabel

**Affiliations:** a Department of Psychology, Oakland University, Rochester, MI, USA; Department of Psychology, University of Western Ontario, London, Canada

**Keywords:** humor, personality, PID-5, pathology, dark

## Abstract

Basic personality traits (e.g., extraversion) have been found to be associated with the humor styles that individuals employ. In the present study, we were interested in determining whether pathological personality traits were also associated with humor styles. We examined the associations between the pathological personality traits captured by the Personality Inventory for the DSM-5 (PID-5) and humor styles in a sample of college students (N = 594). Negative affectivity and detachment were negatively associated with the affiliative and self-enhancing humor styles. Antagonism was positively associated with the aggressive humor style but negatively associated with the affiliative humor style. Disinhibition was positively associated with the aggressive humor style, whereas disinhibition and psychoticism were both positively associated with the self-defeating humor style. Discussion focuses on the implications of these findings and how they can expand our understanding of the connections between the darker aspects of personality and humor.

## Introduction

Researchers have approached the study of humor in a wide variety of ways. For example, classic psychodynamic perspectives concerning humor usually suggested that people employ humor as a defense mechanism to deal with feelings of anxiety or express unconscious desires (e.g., Freud, 1928), whereas more recent theories have suggested that humor serves a variety of intrapsychic and interpersonal functions (see Martin, 2007, for a review). Studies concerning humor have often focused on the relatively positive aspects of humor which include comforting the self or forging social bonds with other people (e.g., Carroll & Shmidt, 1992; Dillon & Totten, 1989; Kuiper, Martin, & Olinger, 1993; Martin & Lefcourt, 1983, Martin & Lefcourt, 1984; Svebak, 1974). Although the positive aspects of humor are certainly important, there are also darker aspects to humor that involve inflicting damage to the self (e.g., belittling one’s own abilities or accomplishments) or harming others (e.g., disparaging a particular person or an entire group; Baron, 1978; Ford & Ferguson, 2004; Greengross & Miller, 2008). The present study will capture the positive and negative aspects of humor by employing the humor styles model developed by Martin and his colleagues (e.g., Martin, Puhlik-Doris, Larsen, Gray, & Weir, 2003) and examine whether these humor styles are associated with pathological personality traits.

Humor is largely an interpersonal phenomenon despite the fact that certain aspects of humor are considered to be important in intrapsychic processes (Martin et al., 2003). In social contexts, humor fulfills various functions including social control, status maintenance, group cohesion, and integration (Martin, 2007). For example, the use of humor has been found to decrease the social distance between individuals during initial encounters which provides a distinct social advantage to those with greater skill at both employing and recognizing humor (Graham, 1995). The framework developed by Martin et al. (2003) suggests that there are two underlying dimensions that are essential for understanding humor. The first of these dimensions concerns the fact that humor can take either a benign or an injurious form. The second dimension reflects the target of enhancement such that humor is believed to either enhance relationships with others or enhance the self. These two underlying humor dimensions combine to form four distinct humor styles that are referred to as *affiliative* humor (benign humor that is used to enhance relationships with others), *self-enhancing* humor (benign humor that is used to enhance the self), *aggressive* humor (injurious humor that is used to enhance the self), and *self-defeating* humor (injurious humor that is used to enhance relationships with others).

Affiliative humor involves the use of humor in social situations as a way to strengthen relationships, increase group cohesion, and reduce tension through strategies such as telling humorous anecdotes or engaging in witty banter in order to put others at ease. An individual who is characterized by the affiliative humor style may say funny things in order to increase interpersonal cohesiveness while maintaining a sense of self-acceptance (Martin et al., 2003; Vaillant, 1977). The self-enhancing humor style refers to the use of humor as a way of coping with stressful events and it has been found to be associated with intrapsychic processes (e.g., Martin et al., 2003). For example, someone who is characterized by the self-enhancing humor style may possess the ability to find humor in unpleasant situations which may, in turn, prevent the person from being overwhelmed by negative emotions.

Aggressive humor is a harmful form of humor that is focused on hurting others by insulting, ridiculing, or teasing them. An individual who is characterized by the aggressive humor style will engage in humor that is likely to have a negative impact on others but may sometimes fail to understand the negative impact of this injurious form of humor (Martin et al., 2003). Self-defeating humor involves the use of excessively self-disparaging humor (e.g., a person making disparaging remarks about his or her own intelligence) in order to ingratiate oneself to others. Individuals who rely on the self-defeating humor style may use humor as a way to avoid underlying issues (e.g., fear of failure; Kubie, 1971; Martin et al., 2003).

A rapidly expanding body of research has shown that the benign and injurious humor styles are differentially related to psychological well-being in the ways that would be expected (see Schermer et al., 2015, for a review). The affiliative and self-enhancing humor styles – which are characterized by the use of benign humor – have been found to have positive correlations with a wide range of outcomes including happiness (Ford, McCreight, & Richardson, 2014), satisfaction with life (Dyck & Holtzman, 2013), resiliency (Cann & Collette, 2014), and social competence (Fitts, Sebby, & Zlokovich, 2009). The affiliative and self-enhancing humor styles have also been shown to have negative associations with outcomes such as trauma-related symptoms (Besser, Weinberg, Zeigler-Hill, Ataria, & Neria, 2015), depressive symptoms (Dyck & Holtzman, 2013; Tucker et al., 2013), neuroticism (Dyck & Holtzman, 2013), and social anxiety (Tucker et al., 2013). In contrast, the aggressive and self-defeating humor styles – which are characterized by the use of injurious humor – have been found to be negatively correlated with happiness (Ford et al., 2014) and positively correlated with depressive symptoms (Tucker et al., 2013). Further, individuals with stable high self-esteem have been found to report relatively low levels of injurious humor compared to individuals with unstable high self-esteem or low self-esteem (Vaughan, Zeigler-Hill, & Arnau, 2014). In addition to these shared associations, the aggressive humor style has been found to be positively correlated with engagement in risky behaviors (Cann & Cann, 2013), whereas the self-defeating humor style has been found to be negatively associated with social competence (Fitts et al., 2009) and perceived social support (Dyck & Holtzman, 2013). Taken together, these results suggest that the affiliative and self-enhancing humor styles are associated with psychological adjustment and positive interpersonal relationships, whereas the aggressive and self-defeating humor styles are linked with poor psychological adjustment and unsatisfying interpersonal relationships.

The connections between the humor styles and personality traits have been examined in previous studies. The affiliative and self-enhancing humor styles have generally been found to be positively associated with the basic personality traits of extraversion, agreeableness, and openness, whereas the aggressive and self-defeating humor styles have often been shown to be negatively correlated with agreeableness and conscientiousness but positively correlated with neuroticism (e.g., Galloway, 2010; Greengross & Miller, 2008; Martin et al., 2003; Saroglou & Scariot, 2002; Vernon, Martin, Schermer, Cherkas, & Spector, 2008; Vernon, Martin, Schermer, & Mackie, 2008; see Mendiburo-Seguel, Páez, & Martínez-Sánchez, 2015, for a review). Further, the humor styles have also been found to be linked with darker aspects of personality. For example, the aggressive and self-defeating humor styles have been linked with psychopathy and Machiavellianism (Martin, Lastuk, Jeffery, Vernon, & Veselka, 2012; Veselka, Schermer, Martin, & Vernon, 2010) as well as borderline personality features (Schermer et al., 2015). However, it is important to note that not all dark personality features are associated with aggressive and self-defeating humor styles because narcissism has been shown to be positively correlated with the use of affiliative humor but it is not associated with aggressive or self-defeating humor (Martin et al., 2012; Veselka et al., 2010).

The goal for the present study was to extend what is known about the connections between humor styles and the darker aspects of personality by examining the broad array of pathological personality traits that were described in Section III (“Emerging Measures and Models” in need of further study) of the *Diagnostic and Statistical Manual of Mental Disorders* (DSM-5; American Psychiatric Association, 2013). This alternative model of personality pathology focuses on maladaptive variants of the Big Five personality dimensions of extraversion, emotional stability, agreeableness, conscientiousness, and openness (Thomas et al., 2013). This alternative model of personality pathology led to the development of the Personality Inventory for the DSM-5 (PID-5; Krueger, Derringer, Markon, Watson, & Skodol, 2012) which is used to capture the following pathological personality traits: *negative affectivity* (i.e., the tendency to experience an array of negative emotions), *detachment* (i.e., characterized by introversion, social isolation, and anhedonia), *antagonism* (i.e., aggressive tendencies accompanied by assertions of dominance and grandiosity), *disinhibition* (i.e., impulsivity and sensation seeking), and *psychoticism* (i.e., a disconnection from reality and a tendency to experience illogical thought patterns). Research concerning the PID-5 is still in its nascent stages but this instrument has already demonstrated its efficacy due to its ability to assess extreme or atypical levels of basic personality traits that are not captured by other instruments. For example, the pathological personality traits captured by the PID-5 have been found to have associations with a wide range of phenomena including interpersonal functioning (Southard, Noser, Pollock, Mercer, & Zeigler-Hill, 2015), moral judgments (Noser et al., 2015), mate retention behaviors (Holden, Roof, McCabe, & Zeigler-Hill, 2015), and aggression (Hopwood et al., 2013).

### Overview and Predictions

The present study examined the associations that the PID-5 pathological personality traits had with the humor styles identified by Martin et al. (2003). Our goal was to use the alternative model of personality pathology presented in the DSM-5 as an organizing framework to gain a clearer understanding of these humor styles. Various studies have suggested that individuals with high levels of pathological personality traits may have problematic interpersonal relationships characterized by aggression, manipulation, and exploitation (e.g., Holden et al., 2015; Strickland, Drislane, Lucy, Krueger, & Patrick, 2013). For example, individuals with high levels of antagonism may lash out against others who have personal desires that conflict with their own goals and desires (Harkness, Reynolds, & Lilienfeld, 2014). This is important because pathological personality traits are often accompanied by interpersonal difficulties and the present study may shed light on the role that humor plays in these difficulties (e.g., humor may be used as a means for dominating or belittling others).

Relatively little is known about the connections between the darker aspects of personality and humor styles so it is important to examine the possibility that the pathological personality traits captured by the PID-5 may be associated with how individuals employ humor. For example, it seemed likely that some of these pathological personality traits – such as antagonism – may be associated with the use of injurious forms of humor. Therefore, we sought to extend previous research concerning both pathological personality traits and humor by assessing the relationship between the PID-5 pathological personality traits and humor styles.

Our approach was conceptually similar to previous studies that have employed the PID-5 in order to represent the distinct trait profiles for constructs such as narcissism and psychopathy (e.g., Strickland et al., 2013; Wright et al., 2013). More specifically, we followed the basic analytical approach adopted by Wright et al. (2013) when examining the connections between narcissism and the PID-5. First, we calculated the zero-order correlations that the PID-5 traits had with the humor styles. Second, we examined the unique associations that the PID-5 traits had with the humor styles because these pathological personality traits are often associated with each other.

The affiliative and self-enhancing humor styles are benign forms of humor that are used to enhance relationships with others or affirm the self, respectively. We expected that negative affectivity, detachment, and antagonism would be negatively associated with these benign humor styles. The rationale for these predictions is that previous studies have shown these benign humor styles to be negatively associated with neuroticism but positively associated with extraversion and agreeableness (e.g., Mendiburo-Seguel et al., 2015). We did not have clear predictions for the associations that disinhibition and psychoticism would have with the benign humor styles but we included these pathological personality traits in the present study for exploratory purposes and reportorial completeness.

The aggressive and self-defeating humor styles are injurious forms of humor that are used to enhance the self or strengthen relationships with others, respectively. We expected that all of the pathological personality traits captured by the PID-5 would be positively associated with these injurious forms of humor. The rationale for these predictions is that previous studies have shown these injurious humor styles to be positively associated with neuroticism, psychopathy, Machiavellianism, and borderline personality features as well as negatively associated with agreeableness and conscientiousness (e.g., Martin et al., 2012; Mendiburo-Seguel et al., 2015; Schermer et al., 2015; Veselka et al., 2010).

## Method

### Participants and Procedures

Participants were 594 undergraduates (148 men, 446 women) enrolled in psychology courses at a university in the Midwestern region of the United States who participated in exchange for partial fulfillment of a research participation requirement. Participants completed measures of pathological personality traits and humor styles – along with other measures that are not relevant to the present study (e.g., self-esteem level) – via a secure website. The mean age of the participants was 20.19 years (*SD* = 3.58) and their racial/ethnic composition was 78% White, 8% Black, 5% Asian, 3% Hispanic, and 6% other.

### Measures

#### Pathological Personality Traits

Pathological personality traits were assessed with the brief form of the *Personality Inventory for the DSM-5* (PID-5-BF; Krueger et al., 2012). The PID-5-BF is a 25-item instrument designed to assess the following five broad pathological personality trait dimensions: *negative affectivity* (5 items; e.g., “I worry about almost everything” [α = .74]), *detachment* (5 items; e.g., “I don’t like to get too close to people” [α = .70]), *antagonism* (5 items; e.g., “I use people to get what I want” [α = .72]), *disinhibition* (5 items; e.g., “People would describe me as reckless” [α = .75]), and *psychoticism* (5 items; e.g., “My thoughts often don’t make sense to others” [α = .77]). Participants were asked to rate how accurately each of the items of the PID-5-BF described them using scales ranging from 0 (*very false or often very false*) to 3 (*very true or often true*).

#### Humor Styles

The Humor Styles Questionnaire (HSQ; Martin et al., 2003) is a 32-item measure of the frequency with which respondents employ benign or injurious styles of humor that are either focused on enhancing the self or enhancing relationships with others. The HSQ yields scores for each of the following four styles of humor: affiliative humor (benign humor that is focused on enhancing relationships with others; e.g., “I laugh and joke a lot with my friends”; α = .83), self-enhancing humor (benign humor that is focused on enhancing the self; e.g., “If I am feeling depressed, I can usually cheer myself up with humor”; α = .76), aggressive humor (injurious humor that is focused on enhancing the self; e.g., “If I don’t like someone, I often use humor or teasing to put them down”; α = .70), and self-defeating humor (injurious humor that is focused on enhancing relationships with others; e.g., “I will often get carried away in putting myself down if it makes my family or friends laugh”; α = .81). Participants were asked to respond to these items on scales ranging from 1 (*totally disagree*) to 7 (*totally agree*). The HSQ has demonstrated adequate discriminant and convergent validity in previous studies (e.g., Martin et al., 2003; Saroglou & Scariot, 2002; see Martin, 2007, for a review) and each humor style had adequate internal consistency in the present study.

## Results

The correlations between the pathological personality traits and humor styles are presented in Table 1. The pathological personality traits generally had negative associations with the affiliative and self-enhancing humor styles and positive associations with the aggressive and self-defeating humor styles. Sex differences emerged for many of the variables that were examined in the present study (see Table 2). Men reported higher levels of pathological personality traits than women on each dimension except for negative affectivity where this pattern was reversed (i.e., women reported higher levels of negative affectivity than were reported by men). Men reported greater reliance on aggressive and self-defeating humor styles than were reported by women. Despite the emergence of these sex differences, it is important to note that sex did not moderate any of the results reported in the following section so these sex differences will not be discussed further.

**Table 1:** Intercorrelations and Descriptive Statistics

Variables	1	2	3	4	5	6	7	8	9
1. Negative Affectivity	—								
2. Detachment	.39***	—							
3. Antagonism	.27***	.39***	—						
4. Disinhibition	.22***	.27***	.41***	—					
5. Psychoticism	.44***	.43***	.40***	.45***	—				
6. Affiliative Humor Style	-.17***	-.26***	-.18***	-.04	-.09*	—			
7. Self-Enhancing Humor Style	-.18***	-.19***	-.13***	-.08*	-.02	.48***	—		
8. Aggressive Humor Style	.08	.13**	.33***	.29***	.22***	-.12**	-.09*	—	
9. Self-Defeating Humor Style	.13**	.15***	.21***	.26***	.29***	-.05	.17***	.36***	—
*M*	1.42	0.72	0.53	0.80	1.02	5.39	4.40	3.44	3.37
*SD*	0.69	0.55	0.50	0.60	0.68	1.01	0.99	0.87	1.11

*p < .05. **p < .01. ***p < .001 (two-tailed tests).

**Table 2:** Sex Differences for Pathological Personality Traits and Humor Styles

Variables	Men		Women		
	*M*	(*SD*)	*M*	(*SD*)	*t*
Negative Affectivity	1.21	0.72	1.49	0.67	-4.36***
Detachment	0.84	0.60	0.68	0.52	3.00**
Antagonism	0.62	0.52	0.50	0.49	2.46*
Disinhibition	0.90	0.62	0.77	0.59	2.37*
Psychoticism	1.12	0.66	0.99	0.68	2.14*
Affiliative Humor Style	5.37	1.11	5.40	0.97	-0.37
Self-Enhancing Humor Style	4.43	0.98	4.39	1.00	0.42
Aggressive Humor Style	3.73	0.83	3.35	0.86	4.65***
Self-Defeating Humor Style	3.56	1.11	3.30	1.11	2.51*

*p < .05. **p < .01. ***p < .001.

We used a series of simultaneous multiple regression analyses to examine the unique associations that pathological personality traits had with aspects of humor. The results of these analyses are presented in Table 3.

**Table 3:** Regressions of Humor Styles on Pathological Personality Traits

Variables	Affiliative Humor Style		Self-Enhancing Humor Style		Aggressive Humor Style		Self-Defeating Humor Style	
	*R^2^*	β	*R^2^*	β	*R^2^*	β	*R^2^*	β
Model	.08***		.07***		.14***		.11***	
Negative Affectivity		-.09*		-.17***		-.05		-.01
Detachment		-.22***		-.16***		-.03		.00
Antagonism		-.12*		-.08		.26***		.07
Disinhibition		.06		-.05		.17***		.14**
Psychoticism		.06		.07		.07		.21***

*p < .05. **p < .01. ***p < .001.

### Affiliative Humor Style

The results of the analysis concerning the affiliative humor style found main effects for negative affectivity (β = -.09, *t* = -1.96, *p* = .05), detachment (β = -.22, *t* = -4.69, *p* < .001), and antagonism (β = -.12, *t* = -2.51, *p* = .01) such that individuals endorsed using more affiliative humor when they reported lower levels of negative affectivity, detachment, and antagonism. No other significant effects emerged for this analysis.

### Self-Enhancing Humor Style

The results of the analysis concerning the self-enhancing humor style revealed effects for negative affectivity (β = -.17, *t* = -3.63, *p* < .001) and detachment (β = -.16, *t* = -3.34, *p* < .001) such that individuals reported employing more self-enhancing humor when they possessed lower levels of negative affectivity and detachment. No other significant effects emerged for this analysis.

### Aggressive Humor Style

The results of the analysis concerning the aggressive humor style revealed associations with antagonism (β = .26, *t* = 5.68, *p* < .001) and disinhibition (β = .17, *t* = 3.85, *p* < .001) such that individuals relied more heavily on aggressive humor when they reported higher levels of antagonism and disinhibition. No other significant effects emerged for this analysis.

### Self-Defeating Humor Style

The results of the analysis concerning the self-defeating humor style revealed effects for disinhibition (β = .14, *t* = 3.02, *p* = .003) and psychoticism (β = .21, *t* = 4.21, *p* < .001) such that individuals employed more self-defeating humor when they reported higher levels of disinhibition and psychoticism. No other significant effects emerged for this analysis.

## Discussion

The purpose of the present study was to examine the connections that the pathological personality traits included in the alternative model of personality pathology that was presented in the DSM-5 had with the humor styles identified by Martin et al. (2003). Previous work has established the connections that basic personality traits have with humor styles such that the benign humor styles (i.e., affiliative and self-enhancing) have generally been found to be positively associated with extraversion, agreeableness, and openness, whereas the injurious humor styles (i.e., aggressive and self-defeating) have often been shown to be negatively correlated with agreeableness and conscientiousness but positively correlated with neuroticism (e.g., Mendiburo-Seguel et al., 2015). Further, the injurious humor styles have also been shown to be associated with some of the darker aspects of personality including psychopathy, Machiavellianism, and borderline personality features (Martin et al., 2012; Schermer et al., 2015; Veselka et al., 2010).

The results of the present study were largely consistent with our predictions such that the pathological personality traits had unique associations with the humor styles. As expected, negative affectivity and detachment had unique negative associations with the benign humor styles (i.e., affiliative and self-enhancing humor styles). This suggests that individuals with high levels of negative affectivity or detachment may be uncomfortable using humor to enhance either themselves or their relationships with others. Although both of these pathological personality traits are negatively associated with benign humor styles, the underlying reasons for these associations may be different. For example, it is possible that individuals with high levels of negative affectivity refrain from using affiliative and self-enhancing forms of humor due to their concerns about how these attempts at humor will be interpreted by others, whereas individuals with high levels of detachment may simply lack the motivation to enhance themselves or their relationships with others through the use of humor. We expected negative affectivity and detachment to also have unique positive associations with the injurious humor styles (i.e., aggressive and self-defeating) but these associations did not emerge.

Negative affectivity had a unique negative association with the affiliative and self-enhancing humor styles. These results are consistent with previous findings showing that the negative emotions experienced by individuals with high levels of negative affectivity (e.g., Krueger et al., 2012) have been found to be negatively associated with interpersonal satisfaction (Ozer & Benet-Martinez, 2006) and positively associated with interpersonal self-consciousness (Cuperman & Ickes, 2009). Moreover, neuroticism has also been found to have a positive association with emotion-focused and avoidance coping strategies (Boyes & French, 2009) which may inhibit individuals with high levels of negative affectivity from successfully employing the self-enhancing humor. Individuals with self-enhancing and affiliative humor styles tend to have a humorous outlook on life and are frequently characterized by positive moods and emotions (Martin et al., 2003), whereas individuals with high levels of negative affectivity are expected to experience frequent negative emotions (Wright et al., 2012) which may inhibit these individuals from successfully employing the benign forms of humor.

Detachment had a unique negative association with affiliative and self-enhancing humor styles. These findings are consistent with previous research showing that individuals with high levels of detachment are interpersonally cold and avoidant (Wright et al., 2012). Further, individuals with high levels of detachment report low levels of communal values and engage in relatively few expressions of warmth (Ackerman & Corretti, 2015). The negative associations between detachment and the benign forms of humor are consistent with the results of these earlier studies (Ackerman & Corretti, 2015; Wright et al., 2012).

Antagonism had a unique negative association with the affiliative humor style and a positive association with the aggressive humor style. These results are consistent with the characterization of antagonistic individuals as being cold, callous, and manipulative in their dealings with others (Hopwood et al., 2013; Southard et al., 2015; Strickland et al., 2013). For example, individuals with high levels of antagonism are relatively unconcerned with how their choices could potentially harm others when making moral decisions (Noser et al., 2015). It is important to note that the present results are consistent with previous studies showing that psychopathy and Machiavellianism – which are antagonistic in nature – are associated with similar humor styles (Veselka et al., 2010). Furthermore, the trait of antagonism is thought to be connected to evolved psychological systems concerning agenda protection (Harkness et al., 2014) which is consistent with the possibility that individuals with high levels of antagonism may employ aggressive humor – and refrain from using affiliative humor – in order to serve their own needs even if doing so is unpleasant for others (e.g., using aggressive forms of humor to dominate or intimidate others).

Disinhibition had unique positive associations with the aggressive and self-defeating humor styles. These results are consistent with previous findings showing that aspects of disinhibition are connected to behaviors that are harmful to the self and others (e.g., Cyders, Coskunpinar, & VanderVeen, in press). Further, individuals with high levels of disinhibition tend to display self-destructive impulsive behaviors while under emotional distress (American Psychiatric Association, 2013) so it seems reasonable that individuals who display these tendencies may also spontaneously engage in forms of humor that are harmful to either themselves or others.

Psychoticism had a unique positive association with the self-defeating humor style but it was not associated with the aggressive humor style. This pattern may be explained, at least in part, by the unusual patterns of behavior that often characterize individuals with high levels of psychoticism and may lend themselves to self-disparaging forms of humor. These results may be considered to be conceptually consistent with those of previous studies showing positive associations between psychoticism and the tendency to engage in physical self-harm (e.g., Hopwood et al., 2013).

The results of this study extend our knowledge concerning the connections between the darker aspects of personality and humor styles. For example, individuals with high levels of antagonism tend to rely on aggressive forms of humor which may play some role in their unsatisfying interpersonal relationships. Further, the tendency for individuals with high levels of antagonism to avoid using affiliative forms of humor may further detract from their interactions with others and deprive them of access to social support.

Although the present study had a number of strengths (e.g., large sample, captured a wide array of pathological personality traits), it is important to acknowledge some of its potential limitations. The first limitation is that the direction of causality between pathological personality traits and humor styles cannot be determined due to the correlational nature of the data. The underlying process model for the present study was that certain pathological personality traits would lead individuals to adopt different humor styles (e.g., antagonistic individuals would choose to employ aggressive forms of humor). However, this causal sequence cannot be established using the present data. For example, it is quite possible that consistently utilizing certain humor styles may have influenced the development of pathological personality traits (e.g., people who adopt aggressive humor styles may develop more antagonistic personality traits) or that a third variable may have impacted the development of both pathological personality traits and humor styles (e.g., common genetic factors, early experiences in a harsh social environment). Future research should attempt to gain a better understanding of the causal links between pathological personality traits and humor styles by using experimental designs or longitudinal studies. The second limitation concerns the generalizability of the present results beyond our undergraduate sample. For example, it is possible that the associations between pathological personality traits and humor styles differ across development periods. The third limitation is that the present study relied exclusively on self-report measures of pathological personality traits and humor styles which make it possible that our findings may have been influenced by socially desirable responding. For example, it is possible that some individuals may have been reluctant to acknowledge their aversive personality traits or tendency to employ injurious forms of humor. Future research would benefit from utilizing strategies that are designed to capture pathological personality traits and humor styles that are not completely reliant on self-report (e.g., observer ratings). The fourth limitation is that the present study had far more female participants than male participants. Sex was included in the analyses but it failed to emerge as a moderator of the effects. This suggests that sex does not moderate the associations that pathological personality traits have with humor styles but it would be helpful if future research attempted to replicate these findings with a sample that had a more even balance of men and women. The fifth limitation is that the pathological personality traits used in the present study capture only a limited range of pathological features of personality. For example, the PID-5 pathological trait of antagonism captures extremely *low* levels of agreeableness but the PID-5 fails to capture extremely *high* levels of agreeableness (e.g., gullibility, self-effacement) which may have unique connections with humor styles (e.g., self-effacement may be positively associated with the self-defeating humor style). Despite these limitations, the results of the present study expand the current understanding of the connections between the darker aspects of personality and humor by showing the links between pathological personality traits and humor styles.

## Conclusion

The results from this study revealed that pathological personality traits were associated with specific humor styles. More specifically, negative affectivity and detachment each had unique negative associations with the affiliative and self-enhancing humor styles. Antagonism was positively associated with the aggressive humor style but negatively associated with the affiliative humor style. Disinhibition had unique positive associations with the aggressive and self-defeating humor styles, whereas psychoticism was positively associated with the self-defeating humor style. Taken together, these results suggest that individuals with pathological personality traits tend to employ humor styles that are harmful to themselves and others and avoid using benign forms of humor that may enhance either themselves or their connections with others. These results suggest the intriguing possibility that humor may play at least some role in the interpersonal difficulties that often accompany pathological personality traits.
